# Supplementary material for: The Association between Prepartum Rumination Time, Activity and Dry Matter Intake and Subclinical Hypocalcemia and Hypomagnesemia in the First 3 Days Postpartum in Holstein Dairy Cows
Source: Animals (Basel). 2023 May 12;13(10):1621. doi: 10.3390/ani13101621 (PMC10215870; doi:10.3390/ani13101621)
Supplement: Supplementary file 1 [file animals-13-01621-s001.zip › animals-2299494-supplementary.pdf]

Supplementary Table S1. Power calculations based on the calculated group mean and SD, with group considered to be cows with a positive disorder status (i.e., having subclinical hypocalcemia or hypomagnesemia) or having a negative disorder status. Power for change in rumination (ΔTDR) and activity (ΔTDA) from -3 days prepartum to calving is based on an ability to detect a difference between groups of 20 min/d. Power calculations for the change in dry matter intake (ΔDMI) from -3 to -1 days prepartum is based on an ability to detect a difference of 1 kg/d.

	Outcome			
**Predictor**	**SCH[1], D0**	**SCH, D3**	**HYM[2], D0**	**HYM, D3**
**ΔTDR**	23.33	25.49	19.31	25.81
**ΔTDA**	42.3	53.18	39.42	54.1
**ΔDMI**	54.22	69.49	55.47	74.34

[1] Subclinical hypocalcemia (SCH) status: Cows were considered subclinical hypocalcemia (SCH+) if plasma Ca 8.6 mg/dL and ≤ 7.8 mg/dL at D0 for primiparous and multiparous cows, respectively and Ca 8.8 at D3 for both primiparous and multiparous cows, otherwise normo-calcemic (SCH−).

[2] Hypomagnesemia (HYM) status: Cows were considered to be hypomagnesemia (HYG+) if blood Mg < 1.8 mg/dL at D0 and at D3 for primiparous and multiparous cows, otherwise normomagnesemia and classified as HYM−.

Supplementary Table S2. Model information for each analysis, specifying the outcome and predictor of interest and the covariates offered and retained for each model. Outcomes of interest are subclinical hypocalcemia status (SCH) and hypomagnesemia status (HYM) at D0 and D3 postpartum. Checks denote when a covariate was retained in the final model, crosses denote when a variable was offered, but excluded, and NA signifies variables that were not offered a specific model.

Model		Disorder Status[1] (n)		Covariates Offered							
Outcome	Predictor	Positive Status	Negative Status	Parity	Baseline TDR, -14 to -7 DIM	Baseline TDA, -14 to -7 DIM	Baseline DMI, -14 to -7 DIM	DMI, 6 d prepartum	Milk yield, 6 d postpartum	DMI, 6 d postpartum	Genotype
Ca, D0	TDR	30	8	✗	✗	NA	NA	✓	✗	✓	✗
Ca, D0	TDA	30	8	✗	NA	✗	NA	✗	✗	✗	✗
Ca, D0	DMI	37	13	✗	NA	NA	✓	NA	✓	✗	✗
Ca, D3	TDR	29	17	✗	✗	NA	NA	✓	✗	✓	✗
Ca, D3	TDA	29	17	✗	NA	✗	NA	✗	✗	✓	✗
Ca, D3	DMI	39	18	✗	NA	NA	✓	NA	✗	✓	✗
Mg, D0	TDR	22	16	✗	✗	NA	NA	✓	✗	✓	✗
Mg, D0	TDA	22	16	✗	NA	✗	NA	✗	✗	✗	✗
Mg, D0	DMI	30	19	✗	NA	NA	✓	NA	✓	✗	✗
Mg, D3	TDR	33	13	✗	✗	NA	NA	✓	✗	✓	✗
Mg, D3	TDA	33	13	✗	NA	✗	NA	✗	✗	✓	✗
Mg, D3	DMI	41	16	✗	NA	NA	✓	NA	✗	✓	✗

[1]Disorder status: positive represents cows with subclinical hypocalcemia (SCH+) and hypomagnesemia (HYM+). Negative represents cows with normal calcium (SCH−) and normal magnesium status (HYM−).
